# Cocoa (*Theobroma cacao* L.) Seed Proteins’ Anti-Obesity Potential through Lipase Inhibition Using In Silico, In Vitro and In Vivo Models

**DOI:** 10.3390/foods9101359

**Published:** 2020-09-25

**Authors:** Luis Jorge Coronado-Cáceres, Griselda Rabadán-Chávez, Luis Mojica, Blanca Hernández-Ledesma, Lucía Quevedo-Corona, Eugenia Lugo Cervantes

**Affiliations:** 1 Unidad de Tecnología Alimentaria, Centro de Investigación y Asistencia en Tecnología y Diseño del Estado de Jalisco, Guadalajara CP 44270, Mexico; luisjorgecoronado@gmail.com (L.J.C.-C.); grisrab@gmail.com (G.R.-C.); lmojica@ciatej.mx (L.M.); 2 Instituto de Investigación en Ciencias de la Alimentación (CIAL, CSIC-UAM, CEI UAM+CSIC), Nicolás Cabrera, 28049 Madrid, Spain; 3 Departamento de Fisiología, Escuela Nacional de Ciencias Biológicas, Instituto Politécnico Nacional, Wilfrido Massieu s/n esq. Manuel I. Stampa. Col. Unidad Profesional Adolfo López Mateos CP, 07738 Ciudad de México, Mexico; quevedocorona@hotmail.com

**Keywords:** pancreatic lipase, cocoa proteins, bioactive peptides, fecal lipids, anti-obesity, ADMET, molecular docking

## Abstract

The aim of this study was to determine the pancreatic lipase (PL) inhibitory effect of cocoa protein (CP) hydrolysates (CPH) using in silico and in vitro approaches, and an in vivo high-fat diet (HF) obese rat model. The results showed better theoretical affinity on PL for cocoa peptides EEQR, GGER, QTGVQ, and VSTDVNIE released from vicilin and albumins (−6.5, −6.3, −6.2, and −6.1 kcal/mol, respectively). Absorption, distribution, metabolism, and excretion (ADMET) prediction showed the human intestinal absorption (HIA) capacity of orlistat and eight cocoa peptides, demonstrating that they presented a low probability of toxicity with values lower than 0.6, while the orlistat has a high probability of hepatotoxicity with a mean value of 0.9. CPH (degree of hydrolysis of 55%) inhibited PL with an IC_50_ (concentration needed to inhibit 50% of enzyme activity) value of 1.38 mg/mL. The intragastric administration of 150 mg CP/kg/day to rats increased total lipids and triglycerides excretion in feces, ranging from 11% to 15% compared to the HF-diet. The HF + CP-diet also significantly decreased (*p* < 0.05) the apparent rate of fat absorption compared with the HF group. These results suggest that CP has anti-obesity potential by inhibiting PL, thus helping to prevent the development of non-communicable diseases.

## 1. Introduction

Obesity and obesity-associated diseases have reached epidemic proportions worldwide. According to the World Health Organization (WHO), in 2016, at least 1.9 billion adults showed obesity or overweight, thus being equivalent to more than 25 percent of the world’s population. This situation represents a great challenge for human health at a global level. Several studies suggest that obesity favors the development of non-communicable diseases (NCDs), such as diabetes, cardiovascular disease, and some forms of cancer [1].

A molecular target of much interest in the prevention and treatment of obesity is the pancreatic lipase (PL). This enzyme is responsible for the intestinal digestion of triacylglycerols from the diet, limiting their absorption in the gastrointestinal tract. Thus, this enzyme has become an important therapeutic target in the development of anti-obesity agents [2,3]. Nowadays, the only PL inhibitor approved by the Food and Drug Administration (FDA) is orlistat (tetrahydrolipstatin), recommended for long-term use to reduce the absorption of dietary fat. However, its use has been associated to multiple side effects, including allergic reactions, such as hives, bowel urgency, and gas stomach pain, difficulty in breathing, diarrhea, nausea, vomiting, oily bowel movements, swelling of face, throat, tongue, and rectal pain [4]. Natural compounds derived from foods such as proteins and peptides could have a positive impact on the functions and conditions of human health [5,6]. In the gastrointestinal tract, the peptides are released from the food proteins by digestive enzymes such as pepsin, trypsin, and chymotrypsin. Proteins and peptides produced from vegetal food sources by in vitro digestion have been reported to inhibit PL activity in in vitro and in vivo studies, including soybean [7], cumin seed [8], soy milk [9], bean seeds (*Phaseolus vulgaris* L. var. Eureka) [10], pinto bean (*Phaseolus vulgaris* cv. Pinto) [11], yellow field pea (*Pisum sativum* L.) [12], and millet grains [13]. Thus, peptides from natural origin, as components of the daily diet, could have potential therapeutic use to ameliorate obesity with low side effects.

On the other hand, computational docking methods have been developed to predict surface charge of target molecules and interaction affinities with some possible ligands. These methods can be applied for in silico identification of peptides from food proteins with the ability to block molecular targets [14] related to obesity, such as PL.

Cocoa (*Theobroma cacao* L.) is a Mesoamerican ancestral crop [15] with high commercial value in developing countries. Cocoa proteins (CP) and their hydrolysates have been reported to exert antioxidant and antitumor effects [16,17]. Also, the nutrigenomic effects of CP against white adipose tissue (WAT) dysfunction with reduction in inflammatory factors, triglyceride levels, and non-esterified fatty acids (NEFAs) in serum, as well as decrease in body weight and different WATs have been shown [18]. Also, other investigations have suggested the contribution of CP to the release of flavor precursor peptides [10,19]. However, there are no data about the PL inhibitory effects of CP and its relation to the decrease in body weight. Therefore, the objective of this work was to determine the effect of CP as source of PL peptide inhibitors by in silico and in vitro approaches, and to confirm these results in an in vivo high-fat diet (HF) obese rat model.

## 2. Materials and Methods

### 2.1. Chemicals

Orlistat (tetrahydrolipstatin), 3-(N-Morpholino) propanesulfonic acid (MOPS), dimethyl sulfoxide (DMSO), sodium chloride (NaCl), calcium chloride (CaCl_2_), sodium taurodeoxycholate (NaTDC), tributyrin (TC4), 4-nitrophenyl butyrate (4-NPB), and PL (type II, from porcine pancreas, specific activity = 400 U/mg) were purchased from Sigma Chemical Company (St. Louis, MO, USA). 22 G 1½ hypodermic needles, and total lipid (TL100), total cholesterol, and triglycerides kits (Randox Laboratories: Crumlin, UK) were used.

### 2.2. Molecular Docking of Proteins and Peptides from Theobroma cocoa L.

Protein sequences from *T. cocoa* were obtained from UniProt (http://www.uniprot.org/), obtaining storage proteins 21 kDa Albumin (P32765) and Vicilin (Q43358). Subsequently, hydrolysis was performed in silico using the PeptideCutter server (http://web.expasy.org/peptide_cutter/), in which gastrointestinal digestion was simulated using the enzymes pepsin, trypsin, and chymotrypsin, generating potential peptides that were drawn and three-dimensional (3D)-modeled using the MarvinSketch program (ChemAxon Lts, version 17.10, 2017). The crystal structure of human PL (PDB: 1LPB) was obtained from the Protein Data Bank (https://www.rcsb.org/). The molecular docking methodology used was adapted from Pan [20]. The best-ranked docking pose was obtained according to the binding-energy value. Hydrogen atoms, solvation parameters, and atomic charge were added in the AutoDockTools vina 4.5 software [21] with a grid of 30 × 30 × 30 Å, and a radius of 1 Å. Peptide–enzyme interactions were revised and the potential type of interactions were shown with the Discovery Studio 2019 Client viewer (Accelrys Software Inc., San Diego, CA, USA; Discovery Studio Modeling Environment, Accelrys Software Inc., San Diego, CA, USA, 2016).

### 2.3. Prediction of Adsorption, Distribution, Metabolism, Excretion, and Toxicity (ADMET) by Computational Analysis

Adsorption, Distribution, Metabolism, Excretion, and Toxicity (ADMET) for selected peptides was predicted using the AdmetSAR server, a cheminformatics-based web server and free tool for the prediction of chemical ADMET properties, containing 30 high predictive models, used in chemical and pharmaceutical fields. The peptides were converted to a simplified molecular-input line input specification (SMILES) format using the BIOPEP database (http://www.uwm.edu.pl/biochemia/index.php/en/biopep), orlistat from the PubChem portal (https://pubchem.ncbi.nlm.nih.gov/compound/Orlistat), or using the MarvinSketch program (ChemAxon Lts, version 17.10, 2017). Caco-2 cell permeability (CP2), brain/blood barrier (BBB), human intestinal absorption (HIA), *Salmonella typhimurium* reverse mutation assay (AMES) toxicity, carcinogens, acute oral toxicity, and hepatotoxicity were predicted with the help of the AdmetSAR tool (http://lmmd.ecust.edu.cn/admetsar2/).

### 2.4. Protein Extraction from Theobroma cacao L.

Cocoa variety “Criollo” beans were collected in the Municipality of Tuxtla Chico (Chiapas, Mexico). The seeds were obtained from the pods, the mucilage and coat were removed, and the seeds were lyophilized and stored at −20 °C. The seeds were ground, and the flour was defatted in three phases: 1:15 (*w/v*) flour was dissolved in hexane:chloroform (2:1, *v/v*), with three times magnetic stirring during 90 min, and centrifugation at 4700× *g* for 20 min at 4 °C. Once the supernatant was removed, the pellet was collected and allowed to dry in the extraction hood. The dry pellet was used to obtain the acetone dry powder (AcDP) that was prepared according to Voigt [22]. Then, CP was extracted from AcDP that was dissolved in a solution containing l0 mM Tris-HCl (with 2 mM ethylenediaminetetraacetic acid, EDTA, pH 7.5), 0.5 M NaCl (with 2 mM EDTA and 10 mM Tris-HCl, pH 7.5), and 0.1 N NaOH. The supernatants were collected in each phase, mixed, and precipitated with 6 N HCl (pH 3.4), and centrifuged at 10,000× *g* for 20 min at 4 °C. The final supernatant was discarded, and the pellet was lyophilized and stored at −20 °C until further analysis [23]. The protein content of the sample was 81%.

### 2.5. Simulated Gastrointestinal Digestion of Cocoa Proteins (CP)

In vitro simulated gastrointestinal digestion of CP was performed following the procedure by Mojica and collaborators [23], with some modifications. CP were suspended in water (1:20, *w/v*) and a sequential enzyme digestion was carried out with pepsin at an enzyme/substrate (E/S) ratio of 1:20 (*w/w*) at pH 2.0 for 2 h at 37 °C, followed by incubation with pancreatin at an E/S ratio of 1:20 (*w/w*) at pH 7.5 and 37 °C for 2 h. The hydrolysis was stopped by heating at 75 °C for 20 min, and the resulting CP hydrolysate (CPH) was centrifuged at 20,000× *g* for 15 min at 4 °C. CPH was dialyzed to eliminate salts using 0.1 to 0.5 kDa molecular weight cutoff membranes, and then freeze-dried. CPH were stored at −20 °C until analysis. The hydrolysis degree (HD) was performed as described by Cabra [24] and Mojica [23], with some modifications. DH is expressed as percentage of the dissolved protein after precipitation with 0.2 N trichloroacetic acid (TCA), compared to the total dissolved protein (100%) obtained after complete hydrolysis with 2 N sulfuric acid at 100 °C for 4 h.

### 2.6. Pancreatic Lipase (PL) Inhibition

PL activity was determined using the methodology reported by Mateos [25]. Briefly, 10 mM 4-nitrophenyl was dissolved in tert-butane (buffer A), 100 mM TC4, and in tert-butane (buffer B), and finally, the buffer C (2.5 mM MOPS + 0.5 mM NaTDC + 150 mM NaCl + 6 mM CaCl_2_, pH 7.2). A curve was prepared with CPH at concentrations ranging from 0.01 to 2.5 mg/mL. l.5 mg of porcine PL was diluted in 100 μL of the buffer C, and the step was performed in a 0.6 mL conical tube placed in the vortex, and then centrifuged at 9500× *g* for 5 min. The supernatant was diluted (1:250, *v/v*) with buffer C (Buffer PL). Orlistat was diluted in DMSO at different concentrations (0.01 to 1 mg/mL). In the 96-well microplate, 10 µL of samples were mixed with 10 µL of the buffer PL. For the negative control, 20 µL of buffer (without enzyme) was added. For the positive control, 9 μL of the buffer C was added to 1 μL of orlistat and 10 µL of the buffer PL, and stirred during 1 h at room temperature. Then, in a 15 mL volumetric tube, 9 mL of buffer C, 500 µL of buffer A, and 500 µL of buffer B were added and mixed by vortexing (total 10 mL). Using a multichannel micropipette, 100 µL of the previously prepared solution were added to each well. The reaction was performed at 37 °C using a microplate reader (Tecan Infinite M200 Pro, Salzburg, Austria), and the absorbance was measured at 410 nm every min for 20 min. The IC_50_ (concentration needed to inhibit 50% of enzyme activity) value was determined as a function of different concentrations, and calculated with the help of GraphPad Prisma 8.0.1 software (GraphPad Software Inc., La Jolla, CA, USA).

### 2.7. Animals and Diet

Obesity-induced (Spectra/Por^®^ Biotech CE, MWCO 100–500 D, 31 mm, 20 mm) six-week-old male Wistar rats (180 ± 5 g of body weight) were purchased from the Animal House of Autonomous Metropolitan University, Xochimilco (Mexico City, Ciudad de México, Mexico). Rats were maintained under controlled conditions of humidity (40–60%) and temperature (22 ± 2 °C), with 12 h dark/12 h light cycles. The rats were acclimatized for one week with unlimited access to food and water. A total of 21 rats were used that were randomly divided into three dietary groups (*n* = 7 per group) as follows (Table 1): (i) Standard Diet (STD) (TD.05230; Teklad Global Harlan Laboratories, Inc., Madison, WI, USA), with a nutritional value of 3.1 kcal/g as energy density, (ii) HF-diet (TD.88137; Teklad Global Harlan Laboratories, Inc.), with a total energy content of 4.5 kcal/g, and (iii) HF-diet, supplemented once daily with intragastric administration of 150 mg/kg CP (HF + CP). All of the groups were fed ad libitum with free access to water. Feed consumption was monitored daily, and body weight was measured weekly throughout the experiment. After the experimental period (week 8), rats were anesthetized with pentobarbital sodium (32 mg/kg intraperitoneally) after 12 h fasting [18,26,27,28]. The use of male Wistar rats was approved by the Ethics and Research Committee of the National School of Biological Sciences of National Polytechnic Institute from Mexico. Approval key: CONBIOETICA09CEI03720130520. 

### 2.8. Determination of Lipid Content in Feces

Fecal lipid extraction was performed using the Kraus methodology [29], with some modifications. Samples of feces were collected every two weeks, leaving them to dry in an extraction hood, grounded and sieved. In 15 mL conical polypropylene tubes (one each per rat), 1 g of powdered feces was added to 5 mL normal saline solution, and vortexed for 1 min. Then, 5 mL of chloroform in methanol (2:1, *v/v*) was added and vortexed for 1 min. The suspension was centrifuged at 1000 *g* for 10 min at room temperature. When two liquid phases separated by a solid phase were formed, the lower liquid phase containing the extracted lipids in chloroform:methanol was extracted using a 22 G½ needle. This phase was forced to pass through the wall of the tube, and afterwards, the needle was removed, and the phase was drained from the plastic tube and collected in a glass tube. Samples were evaporated in an extraction hood for 3–4 days. The remaining pellet was resuspended in 1 mL of absolute ethanol and the total lipids, total cholesterol, and triglycerides were determined using available kits according to the provider’s recommendations. The apparent rate of fat absorption was calculated as follows: 

Apparent absorption rate of the fat (%) = 100 − (fecal fat content/dietary fat intake) × 100

### 2.9. Statistical Analysis

Data were expressed as mean values ± standard error of the mean (SEM). One-way analysis of variance (ANOVA) was performed followed by the Holm–Sidak test, using the software GraphPad Prism ver. 6.01 statistical software (GraphPad), for multiple comparisons in all quantitative variables. A value of *p* < 0.05 represents a significant difference. 

## 3. Results

### 3.1. ADMET Prediction and Molecular Docking 

The in silico enzymatic hydrolysis of albumin (21 kDa) and vicilin (60.79 kDa) generated 108 peptide sequences (Supplementary Table S1), from 2 to 13 amino acids, with molecular masses ranged from 216.15 (VV) to 1386.69 Da (ATGQSCPEIVVQR). ADMET prediction was performed using 108 identified peptides and orlistat. The results shown in Supplementary Table S2 elucidated various properties such as BBB, HIA, Caco-2 permeability, Ames mutagenesis, carcinogens, hepatotoxicity, and acute oral toxicity. Table 2 shows these results corresponding to cocoa peptides with the highest probability of adsorption (NVQR, AQMACPHL, VAPAGHAVT, PHHCDAEAI, HSDDDGQIR, TATAVV, LQR, and GTIT), and the lowest probability (APLSPGDV).

The prediction parameters by HIA resulting from the adsorption capacity of orlistat and eight peptides, which sequences were NVQR, AQMACPHL, VAPAGHAVT, PHHCDAEAI, HSDDDGQIR, TATAVV, LQR, and GTIT, were 0.631, 0.658, 0.649, 0.632, 0.702, 0.815, 0.631, and 0.658, respectively. Regarding BBB, all cocoa peptides were shown to have a high probability of permeability with values greater than 0.8 that were higher than that shown by orlistat, except for the peptides APLSPGDV and VAPAGHAVT, which showed a negative probability BBB value. In the prediction of permeability in Caco-2, both peptides and orlistat did not present the ability to permeate through this membrane model. In the prediction of hepatotoxicity, some peptides identified by the in silico hydrolysis of CP presented low probability, with values lower than 0.6, while the value for orlistat was 0.9. In the prediction of Ames mutagenesis and carcinogenesis, the CP-derived peptides and orlistat did not present a positive probability. Orlistat presented 1.4 kg/mol of acute oral toxicity, and lower toxicity compared to most of the analyzed CP peptides, presenting only the peptide TATAVV with a higher value of acute oral toxicity.

**Figure 1:**
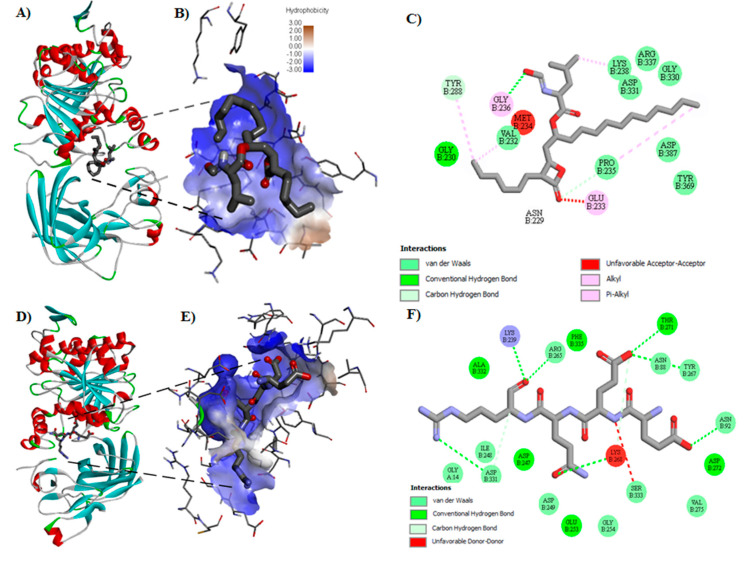
Molecular docking diagrams exemplifying interactions between pancreatic lipase (PL) enzyme (PDB: 1LPB) and inhibitors. (**A**) Example of the pose of orlistat with the enzyme PL. (**B**) The identified pocket of PL interact with orlistat determined by molecular docking. (**C**) Potential interactions with the amino acid residues from the PL catalytic site in two dimensional (2D) with orlistat. (**D**) The best pose of peptide EEQR with the enzyme PL. (**E**) The identified pocket of PL interact with peptide EEQR determined by molecular docking. (**F**) Potential interactions with the amino acid residues from the PL catalytic site in 2D with peptide EEQR.

Molecular docking was carried out to evaluate the affinity of peptides and orlistat for PL. The highest affinity was shown by 15 sequences (Table 3). Peptides EEQR, GGER, QTGVQ, and VSTDVNIE showed the highest theoretical affinity, with values of −6.5, −6.3, −6.2, and −6.1 kcal/mol respectively, that were higher than that shown by orlistat (−4.3 kcal/mol). Figure 1 shows the best representation of the interaction between orlistat and the catalytic site of PL (Figure 1A,B). The drug interaction with several amino acids includes Y_288_ through carbon interaction hydrogen bond, V_232_, K_238_, D_331_, and P_235_ through van der Waals interactions, and E_233_ and G_236_ through alkyl and pi-alkyl interactions (Figure 1C). 

An interaction between PL and EEQR could be observed (Figure 1D,E) with interactions in K_239_, R_265_, T_271_, N_88_, Y_267_, N_92_, S_333_, and D_331_, and double-bond K_268_ presenting interactions of van der Waals, carbon-hydrogen bonds, and unfavorable donor–donor (Figure 1F).

### 3.2. Inhibition of Pancreatic Lipase (PL) by Cocoa Protein (CP) Hydrolysate

**Figure 2:**
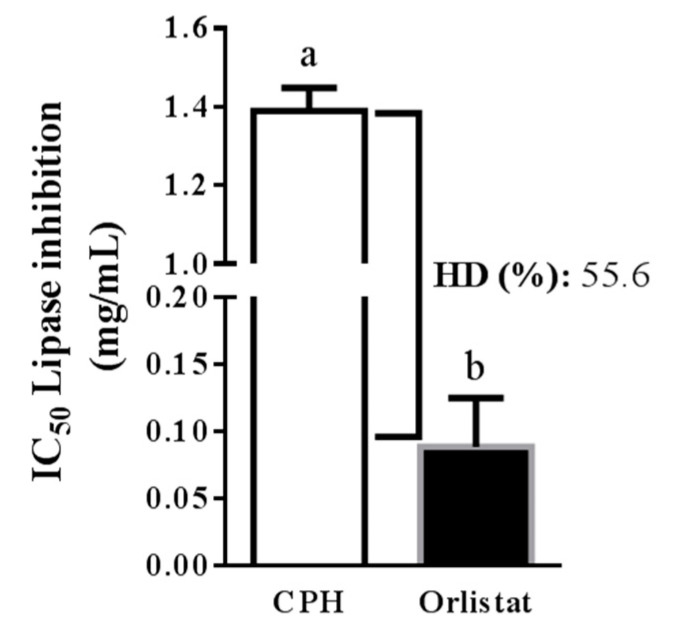
IC_50_ (concentration needed to inhibit 50% of enzyme activity) of cocoa protein hydrolysate (CPH) and orlistat against pancreatic lipase. HD: hydrolysis degree. The data are represented as mean ± standard error of the mean (SEM) of *n* = 3. ^a, b^ indicate significantly different values (*p* < 0.0001).

In this study, the simulation of gastrointestinal digestion of CP was performed, and the potential of CPH to block PL in vitro was examined. Simulated digestion was sequentially performed with pepsin and pancreatin. The HD and PL inhibitory activity (IC_50_) were measured, obtaining values of 55.6% and 1.4 mg protein/mL, respectively. The IC_50_ value measured for orlistat was 0.1 mg/mL (Figure 2). 

### 3.3. Effect of CP in High-Fat Diet-Induced Obese Rats 

**Figure 3:**
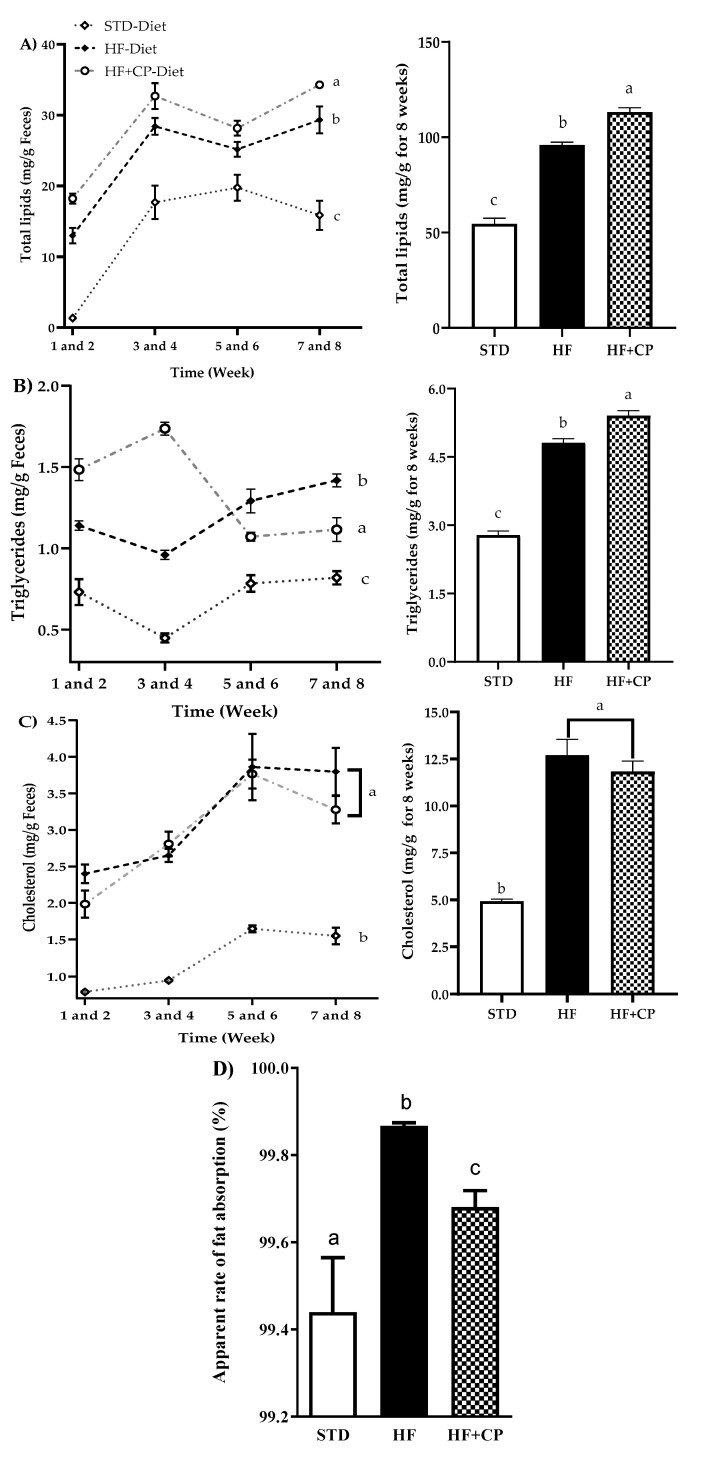
Levels of lipid, cholesterol, and triglycerides in feces for 8 weeks. (**A**) Total lipids in feces for 8 weeks, (**B**) triglycerides, (**C**) cholesterol in feces for 8 weeks, and (**D**) apparent absorption rate of the fat. The data are represented as mean ± standard error of the mean (SEM) (*n* = 7) per group during 8 weeks of treatment. STD: standard diet, HF: high-fat diet, and HF + CP: high-fat diet + 150 mg/kg/day cocoa protein. ^a, b, c^ indicate significantly different values (*p* < 0.05).

The administration of CP (HF + CP group) significantly increased the total lipids and triglycerides content in feces, with a 15% increase in total lipids (Figure 3A) and 11% in triglycerides (Figure 3B). Moreover, a decrease in body weight of the HF + CP group (278.5 ± 6.50 g), in comparison with the HF group (301.6 ± 4.60 g), was observed. Nevertheless, the group of rats fed HF and receiving the CP intragastrically (4102 ± 62.80 kJ) did not show statistically significant differences in total energy intake when compared with the energy intake of the HF group (3992 ± 84.77 kJ). These results may be due to the inhibition of the PL exerted by peptides released during gastrointestinal digestion of CP in rats, resulting in the inhibition of the hydrolysis of triglycerides, and therefore, their excretion. Regarding total cholesterol, there were no significant differences between HF and HF + CP groups (Figure 3C). A significant decrease in the apparent rate of fat absorption in animals fed the HF + CP-diet was observed. This rate was 0.19% lower than that measured in the animals fed the HF-diet, and 0.24% higher than that shown by the STD-diet group (Figure 3D).

## 4. Discussion

There are few studies on the ADMET prediction for peptides from food sources. These studies have mainly evaluated the properties related to absorption and toxicity, simulating a biological model. In our study, the ADMET prediction was made for cocoa peptides by using the AdmetSAR portal. The absorption factor of the cocoa peptides was shown as BBB and HIA, indicating a good probability of adsorption for selected peptides (NVQR, AQMACPHL, VAPAGHAVT, PHHCDAEAI, HSDDDGQIR, TATAVV, LQR, and GTIT). In other studies carried out with fish tripeptides from *Larimichthys crocea* or *Oncorhynchus mykiss*, good absorption BBB and HIA values were also obtained [30]. Another in silico assay with ADMET was performed with soybean tripeptides, highlighting that seven peptides had a good probability of absorption in HIA and four in BBB [31]. Based on the favorable ADMET characteristics, this tool is recommended for future research with peptides and other molecules in the production of functional foods.

The modern lifestyle with little body activity and the excess of triglycerides and free fatty acids from the diet could lead to obesity and dyslipidemia [32]. Among commercially available obesity treatments, one of the most used is that directed to inhibit PL. This enzyme hydrolyzes food triglycerides into monoglycerides and free fatty acids which can be absorbed through epithelial cells in the small intestine and used as an energy source by some tissues or stored in adipose tissue. Thus, PL inhibition results in a decrease of digestion and absorption of triglycerides [33,34]. Besides, the main reason for using PL inhibitors is their action in the gastrointestinal tract, which could reduce some side effects and other health complications associated with current prescribed anti-obesity drugs such as phentermine, diethylpropion, topiramate, and zonisamide [4]. 

The virtual exploration based on the molecular docking by the affinity of molecular targets related to obesity is currently booming, specifically with PL inhibitors [32,35,36]. The results of this work show that after in silico hydrolysis of CP albumin and vicilin, it was possible to generate peptides with potential affinity to interact with human PL (PDB: 1LPB). These positive results provide solid bases for further studies using in vitro and animal models. In silico analyses of PL with food-derived peptides demonstrated that five pinto bean peptides were able to bind porcine PL (PDB: 1ETH) through interactions with catalytic residues of the enzyme, such as S_153_ and H_264_ [11]. Moreover, peptides from *Cuminum cyminum* were able to hinder the catalytic activity of lipase [8]. The use of bioinformatics tools could help to improve the production of new bioactive peptides from food proteins. These peptides might be used in commercial applications to improve food sensory quality (presence of sweet, bitter, umami tastes, and others) and more often, to reduce fat digestion, which has been proven to have a potential impact on human health [37,38]. 

A recent study performed in rats fed a hypercaloric diet has shown that CP decrease body weight gain [18] in spite of consuming the same amount of food, indicating that the decrease in body weight was not due to appetite suppression. The results suggest a potential mechanism involving PL inhibition. Currently, there are several studies on PL inhibitors from different natural origin, but few of them have used food proteins. As examples, peptide fractions of vacuum packaging string bean (*Phaseolus Vulgaris* L.) showed low IC_50_ values (0.05 mg/mL), highlighting an improvement with peptide fraction < 3.5 kDa (IC_50_ = 0.008 mg/mL) [39]. Similarly, protein hydrolysates from *Spirulina platensis*, specifically the 3–5 kDa fraction, show 72% inhibition of PL [40]. Chemically synthesized peptides (at concentration of 20 mg/mL) from cumin seed showed the best PL inhibition, with 54.6% for peptide FFRSKLLSRGAAAAKGALLPQYW, 50.1% for peptide RCMAFLLSDGAAAAQQLLPQYW, and 22.6% for peptide RPAQPNYPWTAVLVFRH [8]. Five peptides identified in pinto beans and chemically synthesized were capable to block PL in an in vitro test, with a maximum inhibition of 86.67% for the peptide LSLEMGSLGALFVCM [11,41]. 

In silico and in vitro PL inhibition results were validated using an animal model of obesity induced by feeding rats an HF-diet, in which lipids present in the feces were evaluated. The results showed a significant increase in fecal fat, when CP were administered to animals, indicating that dietary fats were not absorbed. Similar results were observed in Wistar rats when dietary casein was partially substituted by porcine hemoglobin and porcine globin, with an increase in the excretion of fatty acids in feces [42]. Besides, egg white proteins administered to Wistar rats fed a HF diet and high-sucrose diet increased the excretion of total lipids in the feces, and decreased intestinal fat absorption [43]. The decrease in the apparent rate of fat absorption probably responds to the inhibition of PL, as demonstrated in our previous in vitro and in silico assays. However, the anti-obesity effect could also be due to other factors in the gastrointestinal tract, such as disruption of solubility micellar of cholesterol and chelation of bile acids, as it has been previously demonstrated in other in vitro and in vivo models with proteins and peptides from animal and vegetable origin [6,32,44]. The peptides derived from food proteins offer a natural and safe alternative to reduce obesity and to control obesity-related NCDs. The present report is a pioneer study that suggests the potential of CP as a source of PL inhibitory peptides, resulting in an increase of lipids and total triglycerides in feces from the decrease of fat digestion and absorption rate.

## 5. Conclusions

In the last years, several bioactive components from food sources have been studied, exploring their potential beneficial effects on human health. The anti-obesity effects related to food-derived proteins have become a focus of current attention. The ADMET properties have been predicted for cocoa peptides which were shown to have a good probability of absorption, low probability of hepatotoxicity, and be non-carcinogenic, and non-mutagenic, making it suitable for human consumption. The process of gastrointestinal hydrolysis favors the release of peptides with a potential inhibition of obesity-related molecular targets, specifically PL. CPH was shown to inhibit PL in in silico and in vitro assays. These results were correlated with those obtained from the obese rats fed with the high-fat diet and that received the CP and showed an increase in fecal lipids and a lower apparent rate of fat absorption, which might be the cause, at least in part, of the lower body weight gain. These results suggest that CP has anti-obesity potential by inhibiting PL. Our study underscores the importance of CP and its peptides which could be developed as an ingredient in the formulation of new functional foods intended to mitigate obesity and associated disorders.

## Figures and Tables

**Table 1:** Composition of test diets.

	TD.05230 Standard Diet (STD)	TD.88137 Hypercaloric High-Fat Diet (HF)
Energy (kcal/g)	3.1	4.5
Composition of macronutrients (% by weight)		
Protein	17.3	17.3
Carbohydrate	63.5	48.5
Fat	5.2	21.2
Composition of macronutrients (% kcal)		
Protein	18.7	15.2
Carbohydrate	68.7	42.7
Fat	12.6	42.0
Ingredients (g/kg)		
Casein	195	195
DL-Methionine	3	3
Sucrose	341	341.46
Corn Starch	211.9	150
Maltodextrin	100	-
Anhydrous Milkfat	37.2	210
Cholesterol	12.8	1.5
Cellulose	50	50
Mineral Mix	35	35
Calcium Carbonate	4	4
Vitamin Mix	10	10
Ethoxyquin	0.01	0.04

DL-Methionine: Racemethionine.

**Table 2:** Adsorption, Distribution, Metabolism, Excretion, and Toxicity (ADMET) prediction on orlistat and cocoa peptides.

Molecule	BBB		HIA		CP2		Hepatotoxicity		Ames Mutagenesis		Carcinogenicity		Acute Oral Toxicity
	R	P	R	P	R	P	R	P	R	P	R	P	kg/mol
Orlistat	+	0.592	+	0.973	-	0.561	+	0.9	-	0.8	-	0.829	1.355
NVQR	+	0.969	+	0.631	-	0.893	-	0.5	-	0.7	-	0.757	1.862
AQMACPHL	+	0.777	+	0.658	-	0.866	-	0.525	-	0.8	-	0.714	3.249
VAPAGHAVT	-	0.240	+	0.649	-	0.867	+	0.6	-	0.7	-	0.771	2.609
PHHCDAEAI	+	0.926	+	0.632	-	0.866	+	0.6	-	0.8	-	0.729	2.35
HSDDDGQIR	+	0.955	+	0.702	-	0.863	-	0.6	-	0.7	-	0.757	2.032
TATAVV	+	0.979	+	0.815	-	0.976	-	0.9	-	0.9	-	0.859	1.318
LQR	+	0.972	+	0.631	-	0.889	+	0.525	-	0.7	-	0.757	2.035
GTIT	+	0.777	+	0.658	-	0.866	-	0.525	-	0.8	-	0.714	3.249
APLSPGDV	-	0.237	-	0.547	-	0.863	+	0.625	-	0.8	-	0.743	2.386

Absorption: HIA: Human Intestinal Absorption, CP2: Caco-2 permeability; Distribution: BBB: Blood–brain barrier penetration; Toxicity: Hepatotoxicity, Ames Mutagenesis, Carcinogenesis. A: alanine; C: cysteine; D: aspartic acid; E: glutamic acid; F: phenylalanine; G: Glycine; H: histidine; I: isoleucine; K: lysine; L: leucine; M: methionine; N: Asparagine; P: Proline; Q: glutamine; R: arginine; S: serine; T: threonine; V: valine; Y: Tyrosine; W: tryptophan. Accessed 4 August 2019.

**Table 3:** Results obtained by molecular docking for cocoa peptides and orlistat on the enzyme pancreatic lipase (PL).

Pancreatic Lipase (PDB: ID 1LPB)							
Parent Protein	Digestive Enzyme	Peptide Sequence	Affinity (kcal/mol)	Mass (Da)	Hydrophobicity (kcal/mol)	Net Charge (pH 7.0)	Isoelectric Point
**Vicilin**	Trypsin	EEQR	−6.5	560.25	17.74	−1	4.08
		GGER	−6.3	417.19	15.64	0	6.85
	Pepsin	TIAV	−6	402.24	7.07	0	5.52
		AGRP	−5.9	399.22	11.50	1	11.18
		VTDG	−5.8	390.17	12.48	−1	3.13
	Trypsin	NTQR	−5.8	517.26	11.58	1	10.6
		EQCQR	−5.7	662.27	14.86	0	6.16
	Pepsin	VTDG	−5.8	390.17	12.48	−1	3.13
		NQGAI	−5.6	501.25	10.05	0	5.36
**Albumin**	Pepsin	QTGVQ	−6.2	531.26	10.38	0	5.35
		VSTDVNIE	−6.1	875.42	14.69	−2	2.87
	Trypsin	HSDDDGQIR	−5.9	1041.44	24.22	−2	4.20
	Pepsin	SDNE	−5.7	463.15	16.48	−2	2.87
		CSTSTV	−5.5	596.24	8.84	0	5.25
**Orlistat**	-	-	−4.3	-	-	-	-

A: alanine; C: cysteine; D: aspartic acid; E: glutamic acid; F: phenylalanine; G: Glycine; H: histidine; I: isoleucine; K: lysine; L: leucine; M: methionine; N: Asparagine; P: Proline; Q: glutamine; R: arginine; S: serine; T: threonine; V: valine; Y: Tyrosine; W: tryptophan.

## Supplementary Materials

The following are available online at https://www.mdpi.com/2304-8158/9/10/1359/s1, Table S1: Generation of peptides by in silico hydrolysis from cocoa storage proteins. Table S2. Absorption, distribution, metabolism, excretion (ADMET) prediction on cocoa peptides.
