# Supplementary material for: Cocoa (Theobroma cacao L.) Seed Proteins’ Anti-Obesity Potential through Lipase Inhibition Using In Silico, In Vitro and In Vivo Models
Source: Foods. 2020 Sep 25;9(10):1359. doi: 10.3390/foods9101359 (PMC7599879; doi:10.3390/foods9101359)
Supplement: Supplementary file 1 [file foods-09-01359-s001.pdf]

Supplementary Table S1. Generation of peptides by *in silico* hydrolysis from cocoa storage proteins.

N°	Parent protein	Digestive enzyme	Peptide sequence	Mass (Da)	Hydrophobicity (kcal/mol)	Net Charge (pH 7)	Isoelectric Point
1	Vicilin	Trypsin	CQEQQQGQR	1103.47	18.32	0	6.18
2			SEEEEGQQR	1090.45	27.38	-3	3.61
3			AISQQATSPR	1057.55	12.44	1	11.18
4			CESEATEER	1052.40	25.42	-3	3.61
5			AENSPPLK	854.44	15.17	0	6.86
6			NAQNNQR	843.39	14.3	1	10.60
7			AKPEQIR	840.48	16.43	1	10.20
8			EQCQR	662.27	14.86	0	6.16
9			MVISK	576.32	8.91	1	9.91
10			EEQR	560.25	17.74	-1	4.08
11			EQER	560.25	17.74	-1	4.08
12			EQQR	559.27	14.88	0	6.72
13			HNHK	534.26	16.21	1	9.94
14			VTNGK	517.28	12.49	1	10.14
15			NTQR	517.26	11.58	1	10.6
16			NVQR	515.28	10.87	1	10.60
17			QDR	417.20	14.12	0	6.42
18			GGER	417.20	15.64	0	6.51
19			GGER	417.19	15.64	0	6.85
20			LQR	415.49	9.23	1	10.73
21			CER	406.46	13.32	0	6.14
22			QTR	403.44	10.73	1	10.73
23			NHK	397.43	13.88	1	9.8
24			DPR	386.41	13.49	0	6.41
25			NSK	347.37	12.01	1	9.8
26			TVK	346.43	10.49	1	9.8
27			GQK	331.37	12.62	1	9.8
28			HR	311.34	12.04	1	10.73
29			ASK	304.35	11.66	1	9.8
30			ER	303.32	13.34	0	6.5
31			QR	302.33	10.48	1	10.73
32			NR	288.31	10.56	1	10.73
33			EK	275.31	14.33	0	6.53
34							

	**Pepsin**	EQEEESEEET	1237.45	34.79	-7	2.61
35		VVSQDNQEK	1045.50	19.90	-1	4.00
36		PHHCDAEAI	991.41	19.83	-2	5.02
37		QNMDVAVSA	933.42	13.03	-1	3.13
38		PAGNNKPES	912.42	18.42	0	7.05
39		AQMACPHL	869.38	10.20	0	7.17
40		VAPAGHAVT	821.43	12.35	0	7.89
41		QNTNVIK	815.44	11.84	1	9.84
42		QQGQQGM	775.32	12.61	0	5.32
43		APLSPGDV	754.38	12.22	-1	3.15
44		PVNSPGK	697.37	12.98	1	10.59
45		EACPED	662.22	19.42	-3	2.74
46		QQEEE	661.25	20.33	-3	2.82
47		EANPNT	644.27	14.12	-1	3.08
48		DEEGN	562.18	20.80	-3	2.81
49		CSGVSA	522.21	9.99	0	5.23
50		NQGAI	501.25	10.05	0	5.36
51		LNAVA	486.27	8.04	0	5.6
52		TIAV	402.24	7.07	0	5.52
53		AGRP	399.22	11.50	1	11.18
54		VTDG	390.17	12.48	-1	3.13
55		EEI	389.41	14.04	-2	2.92
56		DQP	358.35	12.45	-1	2.95
57		ETV	347.37	11.32	-1	3.09
58		NNP	343.34	9.74	0	5.44
59		AIN	316.36	8.13	0	5.38
60		EQ	275.26	12.3	-1	3.09
61		EL	260.29	10.28	-1	3.09
62		EV	246.26	11.07	-1	3.09
63		IL	244.33	5.53	0	5.57
64		IV	230.31	6.32	0	5.57
65		AM	220.29	7.73	0	5.53
66		VV	216.28	6.98	0	5.63
67		GE	204.18	12.68	-1	3.21
68		AL	202.25	7.15	0	5.6
69		GEHENY	747.28	18.78	-2	4.08
70	**Chymotrypsin**	SQSPVY	679.31	8.56	0	5.38
71		VPHY	514.58	9.2	0	7.6

72			QQY	437.45	8.73	0	5.36
73			ESY	397.39	11.28	-1	3.09
74			SPF	349.39	6.79	0	5.4
75			CW	307.37	5.79	0	4.94
76			SF	252.27	6.65	0	5.4
77	Albumin 21kDa	Trypsin	ATGQSCPEIVVQR	1386.69	15.32	0	6.17
78			HSDDDGQIR	1041.44	24.22	-2	4.20
79			VTTDGVK	718.38	15.07	0	6.71
80			QVVNAK	657.37	11.90	1	9.84
81			CSDIGR	649.28	13.82	0	6.09
82			SNADSK	620.27	16.61	0	6.51
83			DDVVK	574.29	17.06	-1	3.92
84			VPIR	483.31	8.27	1	11.11
85			IEK	388.46	13.21	0	6.53
86			TIK	360.45	9.83	1	9.8
87			TSK	334.37	11.41	1	9.8
88			ASK	304.35	11.66	1	9.8
89			DR	289.29	13.35	0	6.41
90			MK	277.38	10.03	1	9.8
91			GR	231.26	10.86	1	10.73
92		Pepsin	CPSVCDSCTT	1014.34	12.58	-1	3.00
93			GVANAANSPVL	1011.53	10.68	0	5.58
94			VSTDVNIE	875.42	14.69	-2	2.87
95			SSISGAGGGG	748.33	14.41	0	5.51
96			DNGTPVI	714.35	12.35	-1	3.11
97			GEPGPNT	670.29	15.21	-1	3.08
98			DTDGDE	650.20	23.85	-4	2.68
99			CSTSTV	596.24	8.84	0	5.25
100			DNSAGK	590.26	17.30	0	6.77
101			TATAVV	560.31	8.48	0	5.52
102			QTGVQ	531.26	10.38	0	5.35
103			SDNE	463.15	16.48	-2	2.87
104			SDL	333.34	10.75	-1	3.05
105			DN	247.21	12.39	-1	2.95
106			AGV	245.28	9.09	0	5.6
107			CS	208.23	8.34	0	4.93
108			AL	202.25	7.15	0	5.6

Supplementary Table S2. Absorption, distribution, metabolism, excretion (ADMET) prediction on cocoa peptides.

Molecule	BBB		HIA		CP2		Hepatotoxicity		Ames mutagenesis		Carcinogenicity		Acute Oral Toxicity
	R	P	R	P	R	P	R	P	R	P	R	P	kg/mol
Orlistat	+	0.59	+	0.97	-	0.56	+	0.9	-	0.8	-	0.83	1.36
VTNGK	+	0.95	-	0.42	-	0.90	+	0.53	-	0.6	-	0.84	1.76
CQEQQQGQR	+	0.97	-	0.60	-	0.86	-	0.73	-	0.6	-	0.80	1.55
EEQR	+	0.97	-	0.63	-	0.91	-	0.58	-	0.8	-	0.80	1.33
EQER	+	0.97	-	0.63	-	0.92	-	0.50	-	0.8	-	0.80	1.33
EQCQR	+	0.97	-	0.60	-	0.89	+	0.53	-	0.7	-	0.80	1.70
SEEEEGQQR	+	0.97	-	0.54	-	0.86	-	0.73	-	0.7	-	0.83	1.39
CESEATEER	+	0.97	-	0.57	-	0.86	-	0.65	-	0.7	-	0.73	1.42
AKPEQIR	+	0.91	-	0.43	-	0.86	-	0.68	-	0.7	-	0.74	2.54
AISQQATSPR	+	0.94	-	0.50	-	0.86	-	0.68	-	0.7	-	0.76	2.51
AENSPPLK	+	0.81	-	0.50	-	0.86	-	0.65	-	0.8	-	0.76	2.46
NAQNNQR	+	0.97	-	0.54	-	0.87	-	0.53	-	0.8	-	0.76	1.39
EQQR	+	0.97	-	0.63	-	0.91	-	0.53	-	0.8	-	0.80	1.31
GGER	+	0.96	-	0.69	-	0.94	-	0.55	-	0.7	-	0.79	1.71
HNHK	+	0.95	-	0.50	-	0.92	-	0.50	-	0.7	-	0.79	1.82
MVISK	+	0.96	-	0.43	-	0.86	+	0.60	-	0.7	-	0.77	2.55
NTQR	+	0.97	-	0.52	-	0.91	-	0.55	-	0.7	-	0.73	1.57
NVQR	+	0.97	+	0.63	-	0.89	-	0.50	-	0.7	-	0.76	1.86
AGRP	+	0.92	-	0.65	-	0.89	-	0.65	-	0.6	-	0.69	1.73
APLSPGDV	-	0.24	-	0.55	-	0.86	+	0.63	-	0.8	-	0.74	2.39
AQMACPHL	+	0.78	+	0.66	-	0.87	-	0.53	-	0.8	-	0.71	3.25
CSGVSA	+	0.94	-	0.44	-	0.87	+	0.58	-	0.5	-	0.67	2.24
DEEGN	+	0.96	-	0.69	-	0.91	-	0.50	-	0.6	-	0.74	1.07
EACPED	+	0.81	-	0.72	-	0.88	-	0.53	-	0.8	-	0.64	2.30
EANPNT	+	0.83	-	0.68	-	0.88	+	0.68	-	0.8	-	0.71	2.16
EQEEESEEET	+	0.96	-	0.59	-	0.86	-	0.68	-	0.8	-	0.71	1.54
VVSQDNQEK	+	0.95	-	0.39	-	0.86	-	0.63	-	0.8	-	0.81	2.03
VAPAGHAVT	-	0.24	+	0.65	-	0.87	+	0.60	-	0.7	-	0.77	2.61
TIAV	+	0.97	-	0.45	-	0.89	-	0.50	-	0.8	-	0.84	3.08
VTDG	+	0.94	-	0.46	-	0.91	+	0.53	-	0.7	-	0.83	1.43
QNMDVAVSA	+	0.96	-	0.40	-	0.86	+	0.53	-	0.7	-	0.79	2.17
PAGNNKPES	+	0.85	-	0.66	-	0.86	-	0.65	-	0.7	-	0.69	2.08
PHHCDAEAI	+	0.93	+	0.63	-	0.87	+	0.60	-	0.8	-	0.73	2.35
NQGAI	+	0.97	-	0.39	-	0.89	+	0.63	-	0.7	-	0.76	2.25
LNAVA	+	0.95	-	0.39	-	0.83	+	0.53	-	0.8	-	0.76	1.51
QQEEE	+	0.96	-	0.42	-	0.87	-	0.58	-	0.8	-	0.83	2.31

QNTNVIK	+	0.96	-	0.42	-	0.87	-	0.58	-	0.8	-	0.83	0.83
PVNSPGK	+	0.81	-	0.51	-	0.87	+	0.55	-	0.8	-	0.76	2.03
VTTDGVK	+	0.95	-	0.46	-	0.87	+	0.70	-	0.7	-	0.83	1.98
VPIR	+	0.88	-	0.47	-	0.86	-	0.60	-	0.7	-	0.70	2.77
CSDIGR	+	0.97	-	0.40	-	0.88	+	0.58		0.7	-	0.80	1.86
SNADSK	+	0.94	-	0.53	-	0.89	+	0.60	-	0.8	-	0.73	1.28
DDVVK	+	0.96	-	0.43	-	0.88	-	0.55	-	0.8	-	0.74	1.77
HSDDDGQIR	+	0.96	+	0.70	-	0.86	-	0.60	-	0.7	-	0.76	2.03
QVVNAK	+	0.96	-	0.39	-	0.87	+	0.80	-	0.7	-	0.76	2.23
ATGQSCPEIVVQR	+	0.94	-	0.49	-	0.86	-	0.58	-	0.6	-	0.76	2.91
SSISGAGGGG	+	0.94	-	0.42	-	0.86	+	0.55	-	0.7	-	0.81	2.38
VSTDVNIE	+	0.96	-	0.44	-	0.86	-	0.55	-	0.8	-	0.81	2.40
GVANAANSPVL	+	0.79	-	0.50	-	0.86	-	0.60	-	0.8	-	0.76	2.30
GEPGPNT	+	0.85	-	0.68	-	0.87	+	0.58	-	0.7	-	0.71	2.42
CSTSTV	+	0.95	-	0.48	-	0.87	-	0.50	-	0.6	-	0.70	2.17
CPSVCDSCTT	+	0.78	-	0.58	-	0.86	-	0.58		0.7		0.77	2.88
DNGTPVI	+	0.81	-	0.53	-	0.87	+	0.58	-	0.7	-	0.79	2.73
DNSAGK	+	0.94	-	0.56	-	0.90	+	0.60	-	0.7	-	0.71	1.16
DTDGDE	+	0.97	-	0.64	-	0.89	-	0.53	-	0.7	-	0.74	1.42
QTGVQ	+	0.96	-	0.42	-	0.90	-	0.68	-	0.6	-	0.83	2.00
SDNE	+	0.95	-	0.59	-	0.93	-	0.58	-	0.7	-	0.81	1.15
TATAVV	+	0.98	+	0.82	-	0.98	-	0.90	-	0.9	-	0.86	1.32
IEK	+	0.97	-	0.43	-	0.88	-	0.68	-	0.8	-	0.74	1.44
TIK	+	0.96	-	0.45		0.87	-	0.55	-	0.7	-	0.84	2.19
TSK	+	0.94	-	0.62		0.92	-	0.63	-	0.8	-	0.73	1.00
SDL	+	0.94	-	0.42	-	0.86	-	0.58	-	0.9	-	0.81	1.04
ASK	+	0.64	-	0.83	-	0.85	-	0.58	-	0.8	-	0.70	1.34
AGV		0.92	-	0.44	-	0.88	-	0.50	-	0.7	-	0.57	2.50
QDR	+	0.97	-	0.63	-	0.94	-	0.58	-	0.7	-	0.80	1.06
GIND	+	0.95	-	0.39	-	0.90	-	0.53	-	0.7	-	0.76	1.60
LQR	+	0.97	+	0.63	-	0.89	+	0.53	-	0.7	-	0.76	2.04
QTR	+	0.97	-	0.65	-	0.91	+	0.53	-	0.6	-	0.79	1.49
NHK	+	0.95	-	0.50	-	0.93	-	0.50	-	0.6	-	0.79	1.62
ESY	+	0.81	-	0.43	-	0.91	+	0.60	-	0.7	-	0.70	1.82
GTIT	+	0.78	+	0.66	-	0.87	-	0.53	-	0.8	-	0.71	3.25
DPR	+	0.94	-	0.71	-	0.91	-	0.50	-	0.7	-	0.73	1.49
DQP	+	0.89	-	0.93	-	0.74	-	0.58	-	0.7	-	0.70	1.11
EEI	+	0.98	-	0.43	-	0.85	-	0.68	-	0.9	-	0.74	1.93
SPF	-	0.31	-	0.58	-	0.81	+	0.60	-	0.8	-	0.69	2.16
NSK	+	0.94	-	0.62	-	0.96	-	0.58	-	0.5	-	0.79	1.20
ETV	+	0.97	-	0.46	-	0.86	-	0.60	-	0.8	-	0.83	1.71
TVK	+	0.96	-	0.45	-	0.91	-	0.55	-	0.7	-	0.84	2.14
NNP	+	0.90	-	0.74	-	0.91	-	0.50	-	0.8	-	0.70	1.17
ATF	+	0.93	-	0.42	-	0.83	-	0.58	-	0.8	-	0.66	1.49

GQK	+	0.96	-	0.69	-	0.93	-	0.65	-	0.5	-	0.74	1.46
AIN	+	0.96	-	0.39	-	0.86	-	0.53	-	0.8	-	0.76	1.71
ASK	+	0.94	-	0.62	-	0.93	-	0.58	-	0.8	-	0.70	1.34
ASA	+	0.95	-	0.61	-	0.92	-	0.50	-	0.8	-	0.57	1.00
DR	+	0.97	-	0.73	-	0.95	-	0.60	-	0.6	-	0.83	0.53
MK	+	0.97	-	0.74	-	0.78	-	0.60	-	0.6	-	0.73	2.04
DN	+	0.96	-	0.74	-	0.95	-	0.58	-	0.66	-	0.79	0.21
GR	+	0.97	-	0.73	-	0.92	-	0.65	-	0.5	-	0.83	1.22
CS	+	0.94	-	0.94	-	0.68	-	0.65	+	0.6	-	0.70	0.73
HR	+	0.96	-	0.50	-	0.88	+	0.60	-	0.6	-	0.83	1.60
QY	+	0.89	-	0.46	-	0.85	-	0.50	-	0.7	-	0.73	1.58
CW	+	0.97	-	0.60	-	0.83	+	0.63	-	0.8	-	0.84	2.08
ER	+	0.97	-	0.73	-	0.93	-	0.73	-	0.8	-	0.83	1.26
QR	+	0.97	-	0.66	-	0.91	-	0.60	-	0.6	-	0.84	1.58
NR	+	0.97	-	0.66	-	0.94	-	0.58	-	0.6	-	0.84	1.27
EK	+	0.97	-	0.81	-	0.94	-	0.68	-	0.7	-	0.77	1.67
EQ	+	0.99	-	0.74	-	0.94	-	0.63	-	0.7	-	0.79	1.38
SR	+	0.96	-	0.62	-	0.91	-	0.73	-	0.6	-	0.83	0.78
EL	+	0.98	-	0.45	-	0.87	-	0.68	-	0.9	-	0.79	1.27
SF	+	0.93	-	0.48	-	0.77	-	0.58	-	0.8	-	0.74	1.29
EV	+	0.98	-	0.45	-	0.83	-	0.55	-	0.9	-	0.79	1.77
IL	+	0.97	-	0.45	-	0.79	-	0.70	-	0.8	-	0.79	2.41
PK	+	0.95	-	0.87	-	0.76	-	0.80	-	0.8	-	0.77	1.57
SQ	+	0.96	-	0.62	-	0.94	-	0.65	-	0.6	-	0.83	1.09
GR	+	0.97	-	0.73	-	0.92	-	0.65	-	0.5	-	0.83	1.22
IV	+	0.98	-	0.45	-	0.73	-	0.58	-	0.8	-	0.79	3.24
AM	+	0.98	-	0.66	-	0.89	-	0.73	-	0.7	-	0.69	1.48
VV	+	0.97	-	0.46	-	0.77	-	0.60	-	0.8	-	0.62	3.13
